# Rapid Cellular Turnover in Adipose Tissue

**DOI:** 10.1371/journal.pone.0017637

**Published:** 2011-03-02

**Authors:** Alessandra Rigamonti, Kristen Brennand, Frank Lau, Chad A. Cowan

**Affiliations:** 1 Department of Stem Cell and Regenerative Biology, Harvard Stem Cell Institute, Harvard University, Boston, Massachusetts, United States of America; 2 Doctorate of Prenatal Science, Fetal Diagnosis and Therapy, University of Milan, Milan, Italy; 3 Salk Institute for Biological Studies, La Jolla, California, United States of America; Pennington Biomedical Research Center, United States of America

## Abstract

It was recently shown that cellular turnover occurs within the human adipocyte population. Through three independent experimental approaches — dilution of an inducible histone 2B-green fluorescent protein (H2BGFP), labeling with the cell cycle marker Ki67 and incorporation of BrdU — we characterized the degree of cellular turnover in murine adipose tissue. We observed rapid turnover of the adipocyte population, finding that 4.8% of preadipocytes are replicating at any time and that between 1–5% of adipocytes are replaced each day. In light of these findings, we suggest that adipose tissue turnover represents a possible new avenue of therapeutic intervention against obesity.

## Introduction

The molecular and cellular processes that regulate fat mass remain unresolved. White adipose tissue is the only tissue in the body that can markedly change its mass after adult size is reached. Indeed, fat mass can range from 2–3% of body weight to as much as 60–70% of body weight in humans [1]. At the cellular level, the source of increased fat mass in obesity is currently attributed to two mechanisms: adipocyte hypertrophy, the process by which pre-existing fat cells increase in size due to an accumulation of lipids, and adipocyte hyperplasia, increase differentiation from preadipocytes [2], [3], [4]. Many now believe that the total number of fat cells present in most individuals is set during adolescence and that changes in fat mass generally reflect increased lipid storage in a fixed number of adipocytes [5], [6], [7].

Adipocyte turnover is now thought to occur throughout adult life. Estimates of adipocyte turnover in humans vary greatly, from a low of 10% per year by analyzing the integration of ^14^C derived from nuclear bomb tests in genomic DNA [7] to a high of greater than 60% per year (0.16–0.29% per day) by ^2^H_2_O long-term labeling [8]. In both human and mice, turnover rates are believed to be substantially higher in preadipocytes than adipocytes, 4.5% and 0.16–0.29% per day, respectively for human [8] and 5% and 1–1.5% per day, respectively, for mice [9].

New adipocytes are thought to arise from a pool of progenitors that has recently been described and may reside adjacent to the adipose vascular tissue [10]. A population of cells can also be purified based on cell surface marker expression and transplanted into lipodystrophic mice to reconstitute fat depots [11]. Preadipocytes are generally considered to be replicative [12], [13] until the onset of a transcription factor cascade drives adipogenesis [14], [15] and causes growth arrest [16]. Adipocytes are thought to represent a terminal stage of differentiation and are widely believed to lack proliferative ability [17]. Though it was recently demonstrated that there is cellular turnover within the human adipocyte population [7], a characterization of the rate of adipose tissue turnover in mammalian fat tissue has not yet been reported.

Through three independent experimental approaches, we characterized the degree of cellular turnover in adipose tissue. First, adipose cells were pulse-labeled with an inducible histone 2B-green fluorescent protein (H2BGFP) and, following a chase period, the dilution of H2BGFP through cell division was measured to facilitate a broad survey of turnover in adipose tissue. Second, to quantify preadipocyte replication, we measured the percentage of cells expressing the cell cycle antigen Ki67. Third, to assay the rate of formation of new adipocytes, we determined the rate of BrdU incorporation in perilipin-positive adipocytes. We observed rapid turnover of the adipocyte population, finding that 4.8% of preadipocytes are replicating at any time and that between 1–5% of adipocytes are replaced each day.

## Results

### Rapid turnover of cells in the adipocyte population

**Figure 1:**
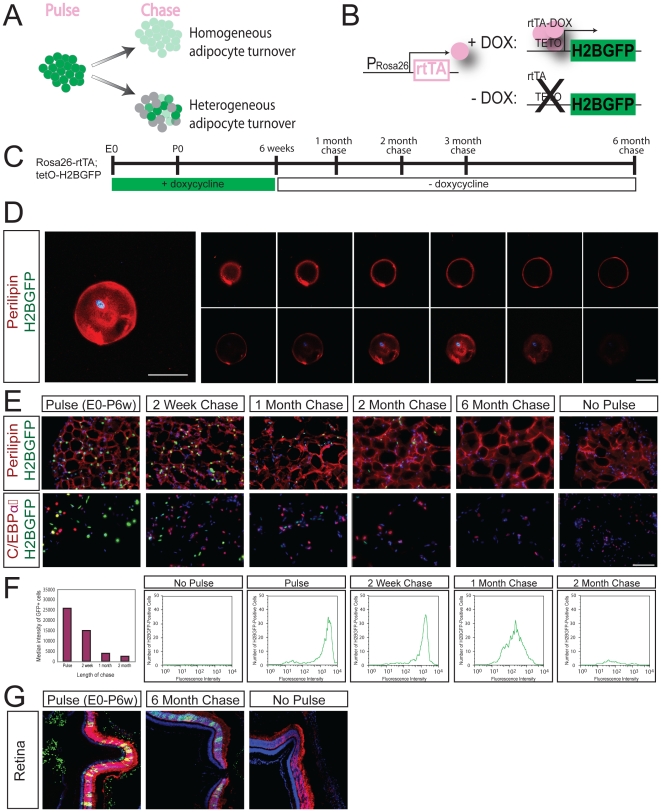
Rapid adipocyte turnover in adipose tissue. **A.** Schematic showing how Rosa26-rtTA; tetO-H2BGFP pulse-chase dilution can distinguish between homogeneous adipocyte turnover (all adipocytes show loss of H2BGFP florescence) and heterogeneous adipocyte turnover (some adipocytes show loss of H2BGFP florescence, others maintain H2BGFP florescence). **B.** Experimental schematic for Rosa26-rtTA; tetO-H2BGFP expression. **C.** Timeline for Rosa26-rtTA; tetO-H2BGFP pulse-chase experiments. **D.** H2BGFP (green) is detected in the nucleus of single murine adipocytes expressing perilipin (red) and DAPI (blue). Scale bars 50 µm. **E.** Uniform loss of label in adult adipocytes following pulse-chase with Rosa26-rtTA; tetO-H2BGFP following a chase period of up to 6 months. Top row, Perilipin (red), DAPI (blue). Bottom row, CEBPα (red), DAPI (blue). No adipocytes are labeled in the absence of pulse. Exposure matched images. Scale bars 100 µm. **F.** FACS histograms of dissociated adipocytes of Rosa26-rtTA; tetO-H2BGFP pulse-chase animals are presented as number of H2BGFP-positive cells versus fluorescence intensity. Median fluorescence intensity of GFP-positive cells versus length of chase shown in graph. **G.** Even after six months of chase, LRCs were detected in the post-mitotic photoreceptor cells of the retina after expression of Rosa26-rtTA; tetO-H2BGFP from E0-6weeks. H2BGFP expression (green), recoverin (red), DAPI (blue). Magnification 200x. All images are exposure matched.

To determine whether all adipocytes turnover or if multiple subpopulations of adipocytes exist, some long lived and others not, the entire adipocyte pool was assayed *in vivo* for label retaining cells (LRCs) (Figure 1A). Tumbar *et al* engineered transgenic mice expressing H2BGFP from a tetracycline-responsive promoter (tetO-H2BGFP) to mark cells and assess their rates of division [18]. To determine the rate of turnover in the adipocyte population, we utilized this inducible H2BGFP strategy in combination with a constitutive promoter that expresses the reverse transactivator (rtTA) within adipose tissue. The Rosa26 locus is active in most cells of the mouse, suggesting that in the presence of doxycycline, Rosa26-rtTA should drive tetO-H2BGFP expression and label most mouse cells, including all the cell types found in adipose depots [19] (Figure 1B). Adipocytes can be identified by expression of two markers: perilipin is a lipid-associated marker of mature differentiated adipocytes [20], [21]; CCAAT/enhancer-binding protein alpha (C/EBPα) is a transcription factor that promotes adipocyte differentiation, is essential for the accumulation of lipids in adipocytes, and is expressed by both adipocytes and preadipocytes, as well as other cell types such as myeloid lineage cells [22], [23]. H2BGFP is diluted with cell division and distributed equally between daughter cells; that all cells, regardless of replicative activity, can be labeled by the inducible H2BGFP system; and that in post-mitotic retinal cell populations, H2BGFP is stable for at least six months [24] (Figure 1G).

To label the adipocyte population with H2BGFP, a group of 40 Rosa26-rtTA; tetO-H2BGFP animals (18 female, 22 male) were pulsed from conception until six weeks of age by administration of doxycycline water. Eight animals (four male, four female) were sacrificed at six weeks of age. All experiments were performed on sibling cohorts. The remaining mice were removed from doxycycline and were sacrificed after chase periods of 1 week (n = 4), 2 weeks (n = 4), 1 month (n = 6), 2 months (n = 6), 3 months (n = 6) and 6 months (n = 6) (Figure 1C). To verify that mature adipocytes were labeled following H2BGFP pulse, we dissociated adipose tissue from pulsed Rosa26-rtTA; tetO-H2BGFP animals and stained individual H2BGFP-positive adipocytes for perilipin (Figure 1D). In sections of pulse-chased Rosa26-rtTA; tetO-H2BGFP adipose tissue, stained with both perilipin and C/EBPα (Figure 1E), we observed uniform loss of H2BGFP label with time.

Due to the presence of large lipid droplets, adipocytes are extremely buoyant and can be easily separated from the other cell types present in dissociated fat tissue based on this physical attribute [10], [25]. In this manner, floating adipocytes can be purified from the nonfloating stromal/vascular (SV) fraction and analyzed by FACS to measure the relative loss of H2BGFP intensity in adipose cells. For these experiments, littermates were pulsed for at least six weeks, and chases were structured so that all animals could be sacrificed and analyzed by FACS on the same day. Comparisons of animals pulsed for 6 or 14 weeks showed no significant increase in the median intensity of the GFP+ population [24]. By analyzing all time points in parallel, we were able to compare the relative GFP intensity between the pulse and chase populations. This experiment was repeated three times using Rosa26-rtTA; tetO-H2BGFP mice and the results were consistent each time. FACS plots of dissociated adipocytes (Figure 1F) indicated that the median intensity of the GFP fluorescence within the adipocyte pool decreased with time. At two months of chase, we typically identify only a few hundred weakly GFP-positive adipocytes in a population of 30,000 cells; if LRCs do exist, they represent less than 1.3±0.7% of the adipocytes examined. We conclude that the diminution of H2BGFP intensity in the adipocyte population is consistent with a model of rapid adipocyte turnover.

### Evidence of replication in adipose tissue

**Figure 2:**
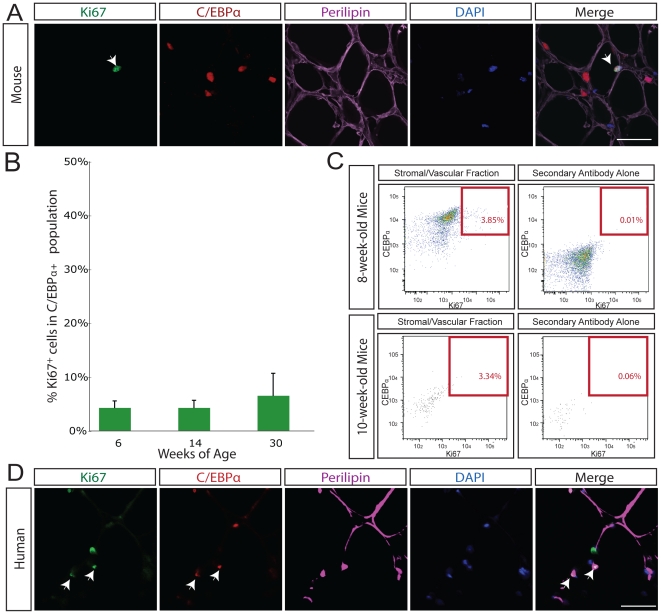
Cellular replication in adipose tissue assayed with the cell cycle protein Ki67. **A.** In mouse adipose tissue, Ki67 (green) overlaps with C/EBPα (red) and DAPI (blue) in perilipin-positive (purple) adipose tissue *in situ*. White arrow indicates triple positive cell. Isotype antibody control shown in right most panel. Scale bars 50 µm. **B.** The percentage of Ki67-positive C/EBPα-expressing cells was determined in 6 (n = 6), 14 (n = 9), and 30-week old (n = 3) mice. Throughout adult life, 4.8% of cells in adipose tissue are Ki67 and C/EBPα-positive at any time. **C.** FACS plots of the stromal/vascular nuclei from dissociated fat tissue of wild-type mice, stained for Ki67 and C/EBPα, as well as appropriate secondary antibody controls. The total percentage of Ki67-positive C/EBPα-positive nuclei in the stromal/vascular fat sample (above secondary alone background for all antibodies) was 3.85%; of this 0.06% represented C/EBPα-high Ki67-positive events, and 3.79% represented C/EBPα-low Ki67-positive events. Top panels represent 8-week-old mice, bottom panels represent 10-week-old mice. **D.** In human adipose tissue, Ki67 (green) overlaps with C/EBPα (red) and DAPI (blue) in perilipin-positive (purple) adipose tissue *in situ*. White arrow indicates triple positive cell. Scale bars 50 µm.

To quantify the percentage of preadipocytes in adipose tissue that are undergoing replication, we first assessed a known marker of cell division within the adipocyte population. The Ki67 antigen is present only in actively dividing cells and is a robust marker of cell replication. It is a nuclear protein found during G1 and S/G2/M phases of the cell cycle but not detectable in quiescent G0 cells [26]. Although the biological function of the antigen remains unresolved, the antigen is essential for maintaining the proliferating state of cells [27]. On stained sections of adipose tissue, we found that Ki67 colocalizes with C/EBPα (Figure 2A) and that 4.8% of cells coexpress CEBPα and Ki67 in 6-week-old C57/BL6 mice (Table S1). There was no significant difference between the percentage of Ki67-positive cells at 6-, 14- or 30-weeks of age (Figure 2B). We observed remarkably little overlap between cells expressing either perilipin or CEBPα with those expressing the vascular endothelial marker PECAM1 (CD31) [28] (Figure S1A) or the macrophage marker Mac1 (Figure S1B,C).

To confirm that a significant fraction of C/EBPα-positive preadipocytes are labeled with Ki67, we analyzed adipose tissue by FACS. The nuclei from adipocytes and SV cells were separated, stained for C/EBPα, Ki67 and the nuclear marker DAPI. The total percentage of Ki67-positive C/EBPα-positive nuclei in the SV fat sample was as high as 3.85% (Figure 2C).

Human adipose tissue also undergoes a significant degree of preadipocyte replication. By confocal microscopy of stained sections of human adipose tissue, we determined the percentage of cells in human adipose tissue expressing Ki67 and found that 0.7% of cells in adipose tissue are positive for Ki67 and C/EBPα at any time (Figure 2D; Table S1).

### BrdU analysis suggests 0.6% of adipocytes are replaced per day

**Figure 3:**
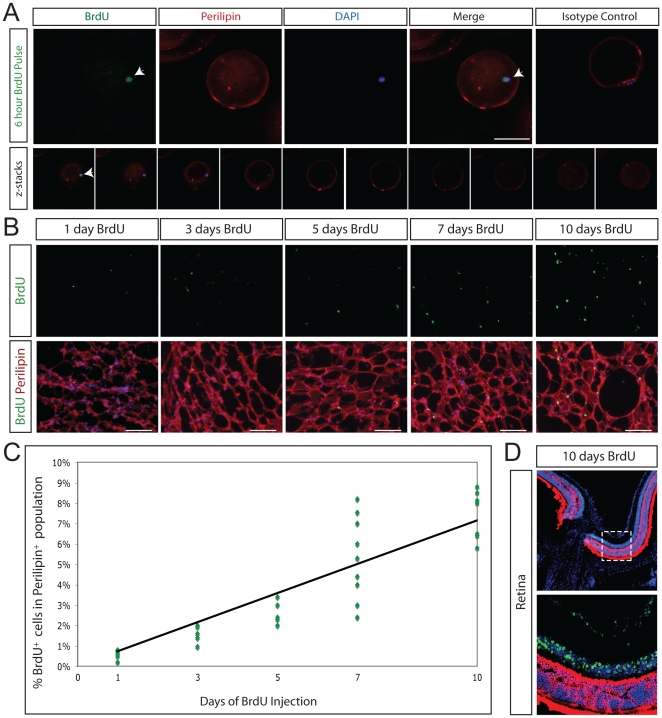
New adipocyte formation assayed with BrdU incorporation. **A, Top.** BrdU incorporation (green) in newly formed single mature adipocyte labeled with perilipin (red) *in situ*. White arrow indicates triple positive cell. BrdU was pulsed for 6 hours. DAPI stained nuclei are blue. Isotype antibody control is shown in right most panel. Scale bars 100 µm. **A, Bottom.** Confocal z-stack images confirming that 6 hours after injection, BrdU incorporation (green) is detected in the nucleus of single murine adipocytes expressing perilipin (red). Magnification, 200x; scale bars 50 µm. **B.** The rate of replication was assayed following daily injection of BrdU into 6-week-old mice for 1 (n = 7), 3 (n = 7), 5 (n = 6), 7 (n = 9) or 10 (n = 7) continuous days. Perilipin staining is shown in red, BrdU staining in green, DAPI stained nuclei are blue. Scale bars 100 µm. **C.** Linear regression analysis of BrdU counts. Each data point on the graph represents the percentage of BrdU-positive cells in the perilipin-positive population from one animal. Rate of new adipocyte formation in 6-week old mice is 1.1% per day. **D.** No BrdU is detected in post-mitotic photoreceptor cells. Recoverin, a marker of photoreceptor cells, is shown in red, BrdU staining in green, DAPI stained nuclei are blue. Top panel, 100x. Bottom panel, 400x. Bottom panel represents the region within the dashed line in the top panel.

Nucleotide base analogs, such as 5-bromo-2′-deoxyuridine (BrdU) are incorporated during DNA synthesis. BrdU labeling of mature adipocytes can result via BrdU incorporation into replicating preadipocytes immediately prior to adipocyte differentiation. Even just 6 hours post BrdU injection *in vivo*, we identified single lipid-filled adipocytes labeled with both BrdU and perilipin (Figure 3A).

To assay the rate of formation of new adipocytes, we determined the rate of BrdU incorporation in perilipin-positive adipocytes in mice at 6-weeks of age (Figure 3B). By varying the duration of BrdU administration, it was possible to plot the number of labeled cells over time and determine a rate of cellular proliferation. The percentage of perilipin-expressing BrdU+ cells was counted for each animal, a linear regression was performed, and adipocyte BrdU incorporation in 6-week old wild-type mice was found to be 0.6% per day (n = 57272 cells) (Figure 3C, Table S1). To ensure that daily BrdU injection did not induce cell damage and permit replication-independent BrdU incorporation, we verified that no BrdU is detected in the post-mitotic mammalian photoreceptor cell population. Photoreceptor cells are located in the outer nuclear layer of the retina and express the calcium-binding protein recoverin [29]; there was no incorporation of BrdU by photoreceptor cells, even following 10 days of continuous BrdU injection (Figure 3D). Each day, 0.6% of the adipocyte population consists of new adipocytes.

Preadipocyte turnover exceeds adipocyte turnover. We assessed BrdU incorporation in the CEBPα-positive population, which includes preadipocytes and adipocytes and found that 1.8% of CEBPα+ cells incorporate BrdU per day (n = 5875 cells). It has long been known that Ki67 labeling is typically greater than BrdU labeling [30], and indeed, in CEBPα-expressing cells, our daily BrdU incorporation rate (1.8%) is less than the growth population determined by Ki67 labeling (4.8%). This likely reflects that BrdU is incorporated only during S-phase of the cell cycle, whereas Ki67 is detected in all phases of mitosis. We find that 1.8% of the combined adipocyte/preadipocyte population incorporates BrdU each day.

### Obese mice show increased adipocyte formation

**Figure 4:**
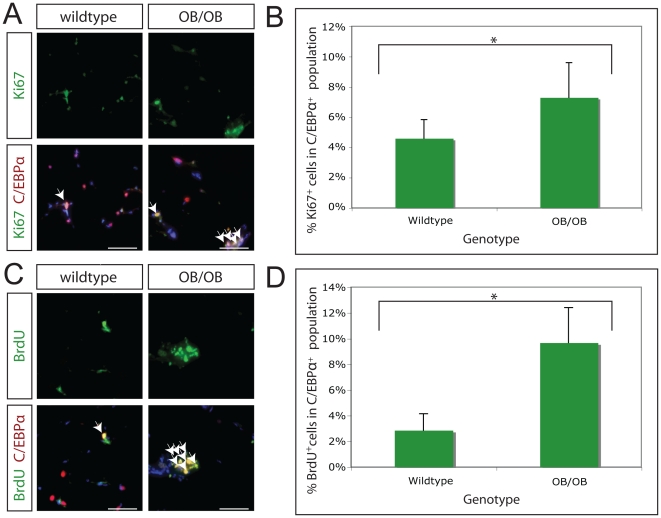
Increased adipose tissue replication in OB/OB mice. **A.** The rate of adipose tissue replication was assayed by co-staining for the cell cycle protein Ki67 (green) and the preadipocyte/adipocyte marker C/EBPα (red) in 14-week-old wildtype and OB/OB mice. Representative images of wildtype and OB/OB adipose tissue are shown. White arrows indicate cells co-staining for Ki67 and C/EBPα. DAPI stained nuclei are blue. Magnification, 200x; scale bars 100 µm. **B.** The percentage of C/EBPα-expressing cells in the growth cycle was quantitated for 14-week old C57/BL6 wildtype (n = 9) and OB/OB (n = 6) mice. There is a statistically significant increase in the rate of preadipocyte replication (p<0.05) from 4.6% to 7.3%, in wildtype and OB/OB mice, respectively. **C.** The rate of adipose tissue replication was assayed by co-staining for BrdU incorporation (green) and the preadipocyte/adipocyte marker C/EBPα (red) in 14-week-old wildtype and OB/OB mice. Representative images of wildtype and OB/OB adipose tissue are shown. White arrows indicate cells co-staining for BrdU and C/EBPα. DAPI stained nuclei are blue. Magnification, 200x; scale bars 100 µm. **D.** The percentage of C/EBPα-expressing cells having incorporated BrdU following 5 days of BrdU injection was quantitated for 14-week old C57/BL6 wildtype (n = 5) and OB/OB (n = 6) mice. There is a statistically significant increase in the rate of adipose tissue replication (p<0.05) from 2.9% to 9.7%, in wildtype and OB/OB mice, respectively.

Finally, we sought to determine whether the rate of adipocyte turnover is altered in obese mice. To do this, we utilized OB/OB knockout mice, in which a mutation in the leptin gene results in profound obesity[31]. Leptin is a hormone expressed predominantly in adipose tissue to modulate body weight and energy expenditure[32]; it is thought to be only expressed in terminally differentiated adipose cells[33], where its expression is regulated by C/EBPα[34]. To ask whether preadipocyte replication is altered in obesity, we determined the percentage of preadipocytes in cell cycle in 14-week-old wildtype (n = 9) and OB/OB (n = 6) mice, all of which were maintained on a C57/BL6 background (Table S1). Ki67 and CEBPα co-expressing adipocyte nuclei were counted for both the wildtype and OB/OB groups, and found to be 4.6% and 7.3%, respectively (Figure 4A,B). This represents a statistically significant increase in preadipocyte replication in OB/OB mice (p<0.05) (Table S1). To confirm increased preadipocyte turnover in OB/OB mice, 14-week-old wildtype (n = 5) and OB/OB (n = 6) mice were injected with BrdU daily for 5 days. BrdU and CEBPα co-labeled adipocyte nuclei were counted for both groups, and BrdU incorporation was found to undergo a significant increase (p<0.001), from 2.9% to 9.7%, in OB/OB mice (Figure 4C,D; Table S1).

## Discussion

In conclusion, significant turnover occurs in adipose tissue. We observed a 40% reduction of median H2BGFP intensity within a two-week chase period, suggesting that the rate of turnover in adipose tissue may approach 5.7% per day. We found evidence for cellular turnover in both the preadipocyte and adipocyte populations. A pulse of BrdU as short as six hours is sufficient to result in labeled mature adipocytes and 0.6% of adipocytes can be labeled with BrdU each day. By cell counting and FACS analysis we observed 4.8% and 3.9%, respectively, of CEBPα-positive cells, representing the combined adipocyte/preadipocyte population, to be positive for Ki67; we also found that 1.8% of CEBPα-positive cells incorporated BrdU per day (Ki67 labeling generally exceeds BrdU incorporation). Therefore, by three independent experimental approaches —dilution of an inducible histone 2B-green fluorescent protein (H2BGFP), labeling with the cell cycle marker Ki67 and incorporation of BrdU — we characterized the degree of cellular turnover in adipose tissue. Our estimate of daily turnover of adipocyte and preadipocyte cells ranges from a low of 1.8% to a high of 5.7%.

Adipocyte turnover is an extremely dynamic process that may provide new opportunities for therapeutic intervention. Although obesity is a metabolic disorder ultimately caused by energy imbalance, a greater understanding of adipose tissue growth and maintenance may one day aid in the treatment of obesity. Our data suggest that adipose tissue undergoes significant turnover, likely driven by preadipocyte replication. In the case of one of the most clinically relevant models of increased fat mass in mice (OB/OB), the rate of preadipocyte replication is significantly increased, supporting the idea that the development of obesity is due to both an increase in fat cell size and fat cell differentiation[2], [3], [4]. This finding may extend to the human population and is consistent with at least one recently published study of human obesity [35]. It could be that changes in adipose tissue that occur in obesity, such as effects on adipokine or oxygen levels, may affect preadipocyte replication and differentiation [35], [36].

We can imagine two time points of therapeutic intervention against preadipocyte replication in cases of human obesity. First, a substantial increase in fat cell number occurs during adolescence and an elevated number of adipocytes at the end of this period is a strong predictor of adult obesity[37], [38]; the ability to slow the rate of preadipocyte replication during this critical period of development may prevent both juvenile and/or adult obesity. Second, if adult obesity itself is caused, or exacerbated, by elevated rates of preadipocyte replication, then targeted intervention to reduce preadipocyte replication in adults may facilitate weight loss in obese patients. We propose that preadipocyte replication may represent a new mechanism for increased fat mass in cases of human obesity and hope that reducing preadipocyte replication may represent a new avenue of therapeutic intervention against obesity.

## Materials and Methods

### Mice

Rosa26-rtTA and tetO-H2BGFP mice were generously provided by Rudolf Jaenisch, Elaine Fuchs, respectively. Rosa26-rtTA and tetO-H2BGFP mice were backcrossed to >95% C57BL/6 inbred background. Mice were first maintained at a barrier facility in the Department of Molecular and Cellular Biology at Harvard University under animal protocol 93-15 and later maintained at a barrier facility at the Centre for Regenerative Medicine, Massachusetts General Hospital, Harvard University under animal protocols 2006N000104 and 2009N000050. Ethics committees at Harvard University and MGH specifically approved all animal work.

### Human Tissue

The ethics committee at Massachusetts General Hospital waived the need for patient consent and specifically approved the human subjects provisions protocol, as the samples obtained were anonymous and would have otherwise been discarded as medical waste in the course of a medically warranted procedure.

### Genotyping

Genotyping was performed by adding a tail biopsy to 100 µl DirectPCR (Viagen) with 30 µg Proteinase K (Roche), incubating overnight at 55°C and denaturing Proteinase K for 20 minutes at 95°C. PCR primers specific to Rosa26-rtTA (A 5′-aaagtcgctctgagttgttat-3′; B 5′-gcgaagagtttgtcctcaacc-3′; C 5′-ggagcgggagaaatggatatg-3′), GFP (forward 5′-ctggtcgagctggacggcgacgtaaac-3′; reverse 5′-atgtgatcgcgcttctcgttgggg-3′) and amplified 300 bp, 600 bp and 600 bp, fragments, respectively. PCR conditions: 95°C for 5 minutes, then 35 cycles of 95°C for 30 seconds, 55°C for 30 seconds, 72°C for 60 seconds, and finally 72°C for 5 minutes.

### Doxycycline and BrdU

Doxycycline (Sigma) was added to drinking water at 1 mg/ml and sweetened with sucrose (1%). Water bottles were changed weekly with freshly prepared solution. Animals were injected with 100 µl of 10 mg/ml BrdU (Sigma) or 300 µl BrdU Labeling Reagent (RPN201, Amersham).

### Immunohistochemistry

Unless otherwise stated, wild-type mice were sacrificed at either 6- or 14-weeks of age. Abdominal fat tissue was dissected from mice, fixed in 4% paraformaldehyde/PBS solution for two hours at 4°C and washed in PBS. Cryo samples were incubated in 30% sucrose/PBS solution overnight, embedded in OCT (Tissue-Tek) and stored at −80°C. Paraffin samples were dehydrated through an ethanol series, washed three times in xylene, embedded in paraffin and stored at 4°C. Frozen samples were sectioned at 30 µm for staining, paraffin samples were sectioned at 20 µm. The following primary antibodies and dilutions were used: rabbit anti-Perilipin A/B (Sigma), 1∶100; goat anti-Perilipin (Novus Biologicals), 1∶100; rabbit anti-CEBPα (Cell Signaling Technology), 1∶50; goat anti-Adipsin (P-16) (Santa Cruz), 1∶200; mouse IgG1 anti-Ki67 (BD PharMingen), 1∶10; rabbit antiKi67 (Abcam), 1∶500; rabbit anti-PH3 (Upstate Cell Signaling), 1∶500; mouse IgG2α anti-PCNA (Calbiochem, NA03), 1∶1000; mouse IgG1 anti-BrdU antibody (clone B44) (BD Bioscience), 1∶50; mouse IgG2α anti-BrdU (Amersham), 1∶200; rat anti-CD31 (BD Bioscience), 1∶100; PE-Cy5 mouse anti-Mac1 (CD11b) (eBioscience), 1∶200; rabbit anti-Recoverin (Chemicon), 1∶2,000. Antigen retrieval was performed for Ki67 and BrdU staining using a Retriever2100 machine (Prestige Medical) in 10 mM citrate buffer (pH 6.0).

Secondary antibodies were Alexa donkey 488, 555, 594 and 647 anti-rabbit (Invitrogen), Alexa goat 488 and 555 anti-mouse IgG1 (Invitrogen), Alexa goat 488 and 555 anti-mouse IgG2α (Invitrogen), Alexa goat 488 and 555 anti-mouse (Invitrogen), Alexa goat 488, 555 and 594 anti-rat (Invitrogen), and Alexa donkey 488, 555, 568 and 594 anti-goat (Invitrogen), all were used at 1∶500. To visualize nuclei, slides were stained with 0.5 µg/ml DAPI (4′,6-diamidino-2-phenylindole) and then mounted with either Aqua polymount (Polyscience, Inc) Mounting Medium or VectaShield Mounting Medium. Images were acquired using a Zeiss LSM510 Meta confocal microscope or a NIKON Eclipse TE2000-E. Ki67 and BrdU cell counts were conducted by epifluorescence microscopy.

Collagen embedding was performed on both floating adipocytes and diluted stromavascular cells (1∶40). In both cases, cells were added to rat collagen type I hydrogel (42% collagen in DMEM), and then, following solidification, fixed in 4% paraformaldehyde for 15 minutes at room temperature and washed with PBS. Embedded adipocytes were immunostained and imaged with a Zeiss LSM510 Meta confocal microscope or a NIKON Eclipse TE2000-E T. Embedded SV cells were stained for 16 hours with Histomark X-gal Substrate (KPL) at 37°C, washed with PBS, stained with DAPI and analyzed at a Leica DM6000B microscope.

Statistical analysis of cell counts was done using a two-tailed unpaired Student's t-test.

### FACS

For sorting of live, unfixed adipose cells, abdominal fat was dissected from mice, rinsed in PBS, and cut with scissors into small pieces of approximately 3 mm in diameter. The fat was then dissociated for 75 minutes in KRB solution (HBSS diluted 1x in PBS; 2% BSA, 12.5 mM HEPES) with 1 mg/ml Collagenase II (Sigma) and 0.2 mg/ml DNAse I (Sigma) at 37°C with high speed shaking. The homogeneous solution was filtered through a 250 µM nylon sieve and centrifuged at 500×g at room temperature for 5 minutes. The upper layer was collected and washed with 50 ml of KRB solution twice by spinning at 500×g for 20 seconds at room temperature. Cells were diluted in KRB 1∶3 and sorted on a BD FACS Calibur (Becton Dickinson).

For sorting of fixed and stained adipose nuclei, abdominal fat was dissected from mice, rinsed in PBS, and cut with scissors into small pieces of approximately 3 mm in diameter. The fat was then dissociated for 75 minutes in KRB solution (HBSS diluted 1x in PBS; 2% BSA, 12.5 mM HEPES) with 1 mg/ml Collagenase II (Sigma) and 0.2 mg/ml DNAse I (Sigma) at 37°C with high speed shaking. The homogeneous solution was filtered through a 250 µM nylon sieve and centrifuged at 500×g at room temperature for 5 minutes. The floating fraction is considered to consist primarily of buoyant adipocytes while the pelleted cells are termed stromal/vascular (SV) cells. Both fractions were collected separately and washed with 50 ml of KRB solution twice by spinning at 500×g for 20 seconds at room temperature.

Nuclei were extracted by incubation 1∶20 in cold lysis buffer (10 mM TrisHCL buffer, 5 mM MgCl2, 0.3 M sucrose and 0.4% NP-20) for 20 minutes on ice, pelleted at 2000×g and washed twice with lysis buffer. They were then layered over a 1 ml cushion of lysis buffer containing 0.88 M sucrose and then collected by centrifugation at 5000×g for 10 minutes. Nuclei were fixed in 4% paraformaldehyde for 15 minutes, pelleted and washed in PBS by centrifugation at 6500×g for 1 minute and finally resuspended in a solution of 0.1% Tween 20 and 0.5% paraformaldehyde in PBS for one hour before being subjected to Ki67 (PE conjugated, BD PharMingen) and C/EBPα immunocytochemistry, stained with DAPI and sorted on a BD FACS LSRII (Becton Dickinson).

## Supporting Information

Figure S1 **Confocal microscopy images demonstrating little overlap between CEBPα and CD31 or MAC1.** **A.** The vascular endothelial marker CD31 (green) overlaps with DAPI (blue) but not with the adipocyte maker perilipin (red) in adipose tissue. Scale bars 100 µm. **B,C.** The macrophage marker Mac1 (green) overlaps with DAPI (blue) but not the adipocyte maker perilipin (red) (**B**) or those cells strongly expressing C/EBPα (red) (**C**) in adipose tissue. Scale bars 100 µm.
(TIF)

Table S1 **Ki67 and BrdU Counts in Adipose Tissue.**
(DOCX)
