# Supplementary figures and images for: Rapid Cellular Turnover in Adipose Tissue
Source: PLoS One. 2011 Mar 2;6(3):e17637. doi: 10.1371/journal.pone.0017637 (PMC3047582; doi:10.1371/journal.pone.0017637)

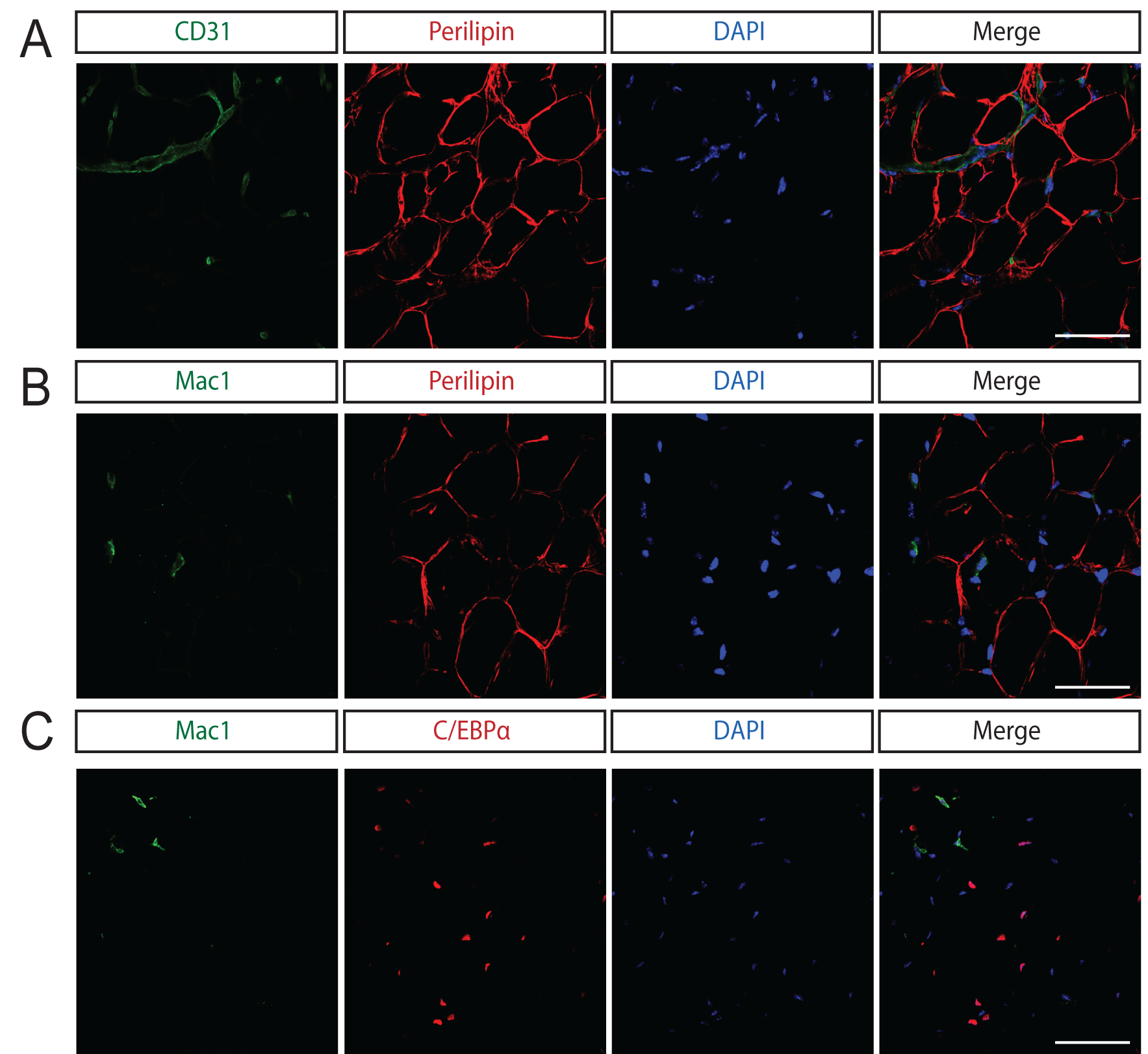

Supplement: Figure S1 — Confocal microscopy images demonstrating little overlap between CEBPα and CD31 or MAC1. A. The vascular endothelial marker CD31 (green) overlaps with DAPI (blue) but not with the adipocyte maker perilipin (red) in adipose tissue. Scale bars 100 µm. B,C. The macrophage marker Mac1 (green) overlaps with DAPI (blue) but not the adipocyte maker perilipin (red) (B) or those cells strongly expressing C/EBPα (red) (C) in adipose tissue. Scale bars 100 µm. (TIF) [file pone.0017637.s001.tif]
