# Supplementary material for: Rapid Cellular Turnover in Adipose Tissue
Source: PLoS One. 2011 Mar 2;6(3):e17637. doi: 10.1371/journal.pone.0017637 (PMC3047582; doi:10.1371/journal.pone.0017637)
Supplement: Table S1 — Ki67 and BrdU Counts in Adipose Tissue. (DOCX) [file pone.0017637.s002.docx]

| **Supplementary Table 1. Ki67 and BrdU Counts in Adipose Tissue** | | | | | | | |
| --- | --- | --- | --- | --- | --- | --- | --- |
| **Cell Replication Marker** | **Species** | **Sex** | **Age** | **Total Nuclei Counted** | **Adipocyte Marker** | **%Cells with Adipocyte Marker** | **Cells Co-Labeled with Ki67** |
| Ki67 | Mouse | Female | 6 weeks | 1169 | CEBPα | 40.5% | 4.0% |
| Ki67 | Mouse | Female | 6 weeks | 1792 | CEBPα | 51.5% | 3.3% |
| Ki67 | Mouse | Female | 6 weeks | 1097 | CEBPα | 54.3% | 4.0% |
| Ki67 | Mouse | Male | 6 weeks | 737 | CEBPα | 44.0% | 3.4% |
| Ki67 | Mouse | Male | 6 weeks | 1834 | CEBPα | 68.0% | 4.2% |
| Ki67 | Mouse | Male | 6 weeks | 1012 | CEBPα | 43.5% | 6.6% |
| Ki67 | Mouse | Female | 14 weeks | 964 | CEBPα | 45.6% | 4.8% |
| Ki67 | Mouse | Female | 14 weeks | 1472 | CEBPα | 44.3% | 4.4% |
| Ki67 | Mouse | Female | 14 weeks | 1092 | CEBPα | 56.7% | 3.6% |
| Ki67 | Mouse | Male | 14 weeks | 1282 | CEBPα | 26.0% | 4.5% |
| Ki67 | Mouse | Male | 14 weeks | 1107 | CEBPα | 48.7% | 2.0% |
| Ki67 | Mouse | Male | 14 weeks | 580 | CEBPα | 39.5% | 6.1% |
| Ki67 | Mouse | Male | 14 weeks | 430 | CEBPα | 58.8% | 4.7% |
| Ki67 | Mouse | Male | 14 weeks | 517 | CEBPα | 56.9% | 5.8% |
| Ki67 | Mouse | Male | 14 weeks | 436 | CEBPα | 61.4% | 5.6% |
| Ki67 | Mouse | Male | 30 weeks | 700 | CEBPα | 35.9% | 11.2% |
| Ki67 | Mouse | Male | 30 weeks | 771 | CEBPα | 60.3% | 9.3% |
| Ki67 | Mouse | Male | 30 weeks | 648 | CEBPα | 54.2% | 3.1% |
| **Ki67** | **Mouse** | **Total** | **Mixed** | **17640** | **CEBPα** | **49.4%** | **5.0%** |
|  |  |  |  |  |  |  |  |
| Ki67 | Human | Female | nd | 2276 | CEBPα | 50.5% | 0.5% |
| Ki67 | Human | Female | nd | 2144 | CEBPα | 45.6% | 1.0% |
| Ki67 | Human | Female | nd | 2298 | CEBPα | 52.9% | 0.7% |
| **Ki67** | **Human** | **Total** | **Mixed** | **6718** | **CEBPα** | **49.7%** | **0.7%** |
|  |  |  |  |  |  |  |  |
| **Cell Replication Marker** | **Species** | **Sex** | **Age** | **Total Nuclei Counted** | **Adipocyte Marker** | **%Cells with BrdU** | **%Cells with BrdU/Day** |
| 1d BrdU | Mouse | Female | 6 weeks | 1756 | Perilipin | 0.5% | 0.5% |
| 1d BrdU | Mouse | Female | 6 weeks | 1512 | Perilipin | 0.2% | 0.2% |
| 1d BrdU | Mouse | Female | 6 weeks | 2921 | Perilipin | 0.8% | 0.8% |
| 1d BrdU | Mouse | Female | 6 weeks | 1007 | Perilipin | 0.2% | 0.2% |
| 1d BrdU | Mouse | Female | 6 weeks | 1190 | Perilipin | 0.6% | 0.6% |
| 1d BrdU | Mouse | Male | 6 weeks | 2438 | Perilipin | 0.8% | 0.8% |
| 1d BrdU | Mouse | Male | 6 weeks | 1433 | Perilipin | 0.7% | 0.7% |
| 3d BrdU | Mouse | Female | 6 weeks | 1862 | Perilipin | 1.9% | 0.6% |
| 3d BrdU | Mouse | Female | 6 weeks | 1580 | Perilipin | 1.6% | 0.5% |
| 3d BrdU | Mouse | Female | 6 weeks | 782 | Perilipin | 1.4% | 0.5% |
| 3d BrdU | Mouse | Female | 6 weeks | 1478 | Perilipin | 2.0% | 0.7% |
| 3d BrdU | Mouse | Female | 6 weeks | 2283 | Perilipin | 1.4% | 0.5% |
| 3d BrdU | Mouse | Male | 6 weeks | 1558 | Perilipin | 1.0% | 0.3% |
| 3d BrdU | Mouse | Male | 6 weeks | 1201 | Perilipin | 2.0% | 0.7% |
| 5d BrdU | Mouse | Female | 6 weeks | 1073 | Perilipin | 3.0% | 0.6% |
| 5d BrdU | Mouse | Female | 6 weeks | 2235 | Perilipin | 2.4% | 0.5% |
| 5d BrdU | Mouse | Female | 6 weeks | 1809 | Perilipin | 2.0% | 0.4% |
| 5d BrdU | Mouse | Female | 6 weeks | 1709 | Perilipin | 3.4% | 0.7% |
| 5d BrdU | Mouse | Male | 6 weeks | 2304 | Perilipin | 2.0% | 0.4% |
| 5d BrdU | Mouse | Male | 6 weeks | 2528 | Perilipin | 2.3% | 0.5% |
| 7d BrdU | Mouse | Female | 6 weeks | 915 | Perilipin | 7.5% | 1.1% |
| 7d BrdU | Mouse | Female | 6 weeks | 1379 | Perilipin | 5.3% | 0.8% |
| 7d BrdU | Mouse | Female | 6 weeks | 2454 | Perilipin | 4.4% | 0.6% |
| 7d BrdU | Mouse | Female | 6 weeks | 1034 | Perilipin | 7.0% | 1.0% |
| 7d BrdU | Mouse | Female | 6 weeks | 779 | Perilipin | 8.2% | 1.2% |
| 7d BrdU | Mouse | Female | 6 weeks | 1489 | Perilipin | 2.4% | 0.3% |
| 7d BrdU | Mouse | Male | 6 weeks | 1053 | Perilipin | 3.0% | 0.4% |
| 7d BrdU | Mouse | Male | 6 weeks | 1051 | Perilipin | 6.0% | 0.9% |
| 7d BrdU | Mouse | Male | 6 weeks | 1502 | Perilipin | 4.0% | 0.6% |
| 10d BrdU | Mouse | Female | 6 weeks | 2622 | Perilipin | 8.1% | 0.8% |
| 10d BrdU | Mouse | Female | 6 weeks | 1719 | Perilipin | 8.0% | 0.8% |
| 10d BrdU | Mouse | Female | 6 weeks | 1160 | Perilipin | 5.8% | 0.6% |
| 10d BrdU | Mouse | Female | 6 weeks | 1035 | Perilipin | 6.4% | 0.6% |
| 10d BrdU | Mouse | Female | 6 weeks | 1258 | Perilipin | 8.8% | 0.9% |
| 10d BrdU | Mouse | Male | 6 weeks | 1238 | Perilipin | 6.5% | 0.7% |
| 10d BrdU | Mouse | Male | 6 weeks | 1925 | Perilipin | 8.5% | 0.9% |
| **Summary** | Mouse | Male | 6 weeks | **57272** | **Perilipin** | **-** | **0.63%** |
| 1d BrdU | Mouse | Male | 6 weeks | 1388 | CEBPα | 2.1% | 2.1% |
| 3d BrdU | Mouse | Male | 6 weeks | 1100 | CEBPα | 4.5% | 1.5% |
| 5d BrdU | Mouse | Male | 6 weeks | 1677 | CEBPα | 8.0% | 1.6% |
| 7d BrdU | Mouse | Male | 6 weeks | 1710 | CEBPα | 14.0% | 2.0% |
| **Summary** | Mouse | Male | 6 weeks | **5875** | **CEBPα** | **-** | **1.78%** |
